# Prevalence of Congenital Malformations in the Oriental Region of Morocco

**DOI:** 10.7759/cureus.88441

**Published:** 2025-07-21

**Authors:** Islam Erraoui, Mohammed Ech-Chebab, Anass Ayyad, Sahar Messaoudi, Rim Amrani

**Affiliations:** 1 Department of Pediatrics, University Hospital Center of Mohammed VI, Faculty of Medicine and Pharmacy, Mohammed Premier University, Oujda, MAR; 2 Department of Neonatology and Neonatal Intensive Care, University Hospital Center of Mohammed VI, Faculty of Medicine and Pharmacy, Mohammed Premier University, Oujda, MAR; 3 Department of Neonatology and Neonatal Resuscitation, University Hospital Center of Mohammed VI, Faculty of Medicine and Pharmacy, Mohammed Premier University, Oujda, MAR; 4 Department of Neonatology, University Hospital Center of Mohammed VI, Faculty of Medicine and Pharmacy, Mohammed Premier University, Oujda, MAR

**Keywords:** birth defects, consanguinity, neonatal morbidity, neonatal mortality, prenatal diagnosis

## Abstract

This retrospective study conducted at the Mohammed VI University Hospital Center in Oujda analyzed clinically visible congenital malformations among neonates admitted over a one-year period. The maternal age profile predominantly included young adults, with a significant proportion of cases linked to consanguinity, highlighting a potential genetic contribution to the occurrence of these anomalies. Most malformations were diagnosed after birth, with congenital heart defects representing the most frequent anomaly, followed by major digestive malformations such as anal atresia and omphalocele. Cases involving multiple malformations were also documented. The neonatal outcomes revealed a concerning mortality rate, reflecting the severity of these congenital conditions. These findings emphasize the urgent need to strengthen prenatal screening programs, implement a national registry for congenital malformations, and enhance public health interventions focused on prevention and early diagnosis to improve neonatal prognosis and reduce morbidity and mortality in this region.

## Introduction

Congenital malformations, also known as congenital anomalies or birth defects, are structural or functional abnormalities that occur during intrauterine life and are present at birth. They encompass a wide spectrum of disorders ranging from minor anatomical variations to severe functional impairments affecting one or more organ systems. Globally, these anomalies represent a significant public health issue due to their increasing incidence and long-term impact on affected individuals and health systems [1].

In both developed and developing countries, congenital malformations have emerged as one of the leading causes of neonatal and infant mortality, particularly when associated with major organ system involvement [2]. According to the World Health Organization, approximately 240,000 newborns die worldwide within the first 28 days of life due to congenital disorders each year, underscoring their critical contribution to early-life mortality and the need for preventive strategies [3].

The etiology of congenital malformations is multifactorial and complex, involving an intricate interplay between genetic predispositions and environmental exposures. These may include chromosomal abnormalities, single-gene mutations, maternal infections, nutritional deficiencies, exposure to teratogens, and other sociodemographic factors [4]. Understanding and identifying these risk factors are essential not only for early diagnosis and management but also for the development of targeted public health interventions aimed at reducing the incidence of congenital anomalies and their associated health burden.

## Materials and methods

This was a retrospective and descriptive study aimed at identifying and characterizing clinically recognizable congenital malformations detected at birth or during the immediate neonatal period. The study was conducted over a 12-month period, from July 2022 to July 2023, in the neonatology department of the Mohammed VI University Hospital Center in Oujda, Morocco. This tertiary care center receives deliveries and neonatal admissions from both urban and rural healthcare facilities across the Oriental region.

All live-born neonates admitted to the department during the study period were evaluated for inclusion. Only those presenting with major or minor congenital malformations that were clinically detectable at birth or within the first 28 days of life were included. Stillbirths, neonates with only functional anomalies (e.g., metabolic disorders), and cases with incomplete or insufficient medical records were excluded from the analysis.

Data were retrospectively collected from paper-based medical records and hospital registries using a standardized data collection form specifically designed for this study. The collected variables included maternal characteristics: age, parity, consanguinity, medical history, and prenatal follow-up; obstetric data: gestational age, mode of delivery, and pregnancy-related complications; and neonatal data: sex, birth weight, type and location of malformations, associated anomalies, and neonatal outcomes.

Descriptive statistical analysis was performed using Microsoft Excel 2019 (Microsoft Corp., Redmond, WA, US). Qualitative variables were expressed as absolute and relative frequencies (percentages).

All figures included in this article are original and based on the data collected during the study. This study was conducted in compliance with institutional ethical standards. Patient anonymity and data confidentiality were strictly maintained throughout the data collection and analysis process.

## Results

During the study period, a total of 368 neonatal admissions were recorded, among which 64 newborns presented with one or more congenital malformations, representing an incidence of 17.3%. The mean maternal age was 29 years, with a range of 17 to 40 years. The mean parity was 2.1 children, ranging from one to five. Consanguinity was identified in 27% of cases, suggesting a possible genetic contribution to the occurrence of these anomalies.

From a medical standpoint, three mothers had gestational diabetes, two had hypothyroidism, and one was being monitored for psychosis during pregnancy. Regarding the timing of diagnosis, the majority of malformations (92.4%) were detected postnatally, while only 7.6% were diagnosed prenatally, indicating a low rate of prenatal detection. Most of the affected newborns were born at term (n = 59), with only five preterm births, suggesting that prematurity was not a predominant factor in this population.

In terms of sex distribution, there was a male predominance (55%), followed by female infants (41%), and 4% of newborns had an undetermined sex at birth. Anthropometric measurements at birth were within normal ranges, with a mean birth weight of 2.81 kg, a mean length of 45.6 cm, and a mean head circumference of 33 cm.

**Figure 1:**
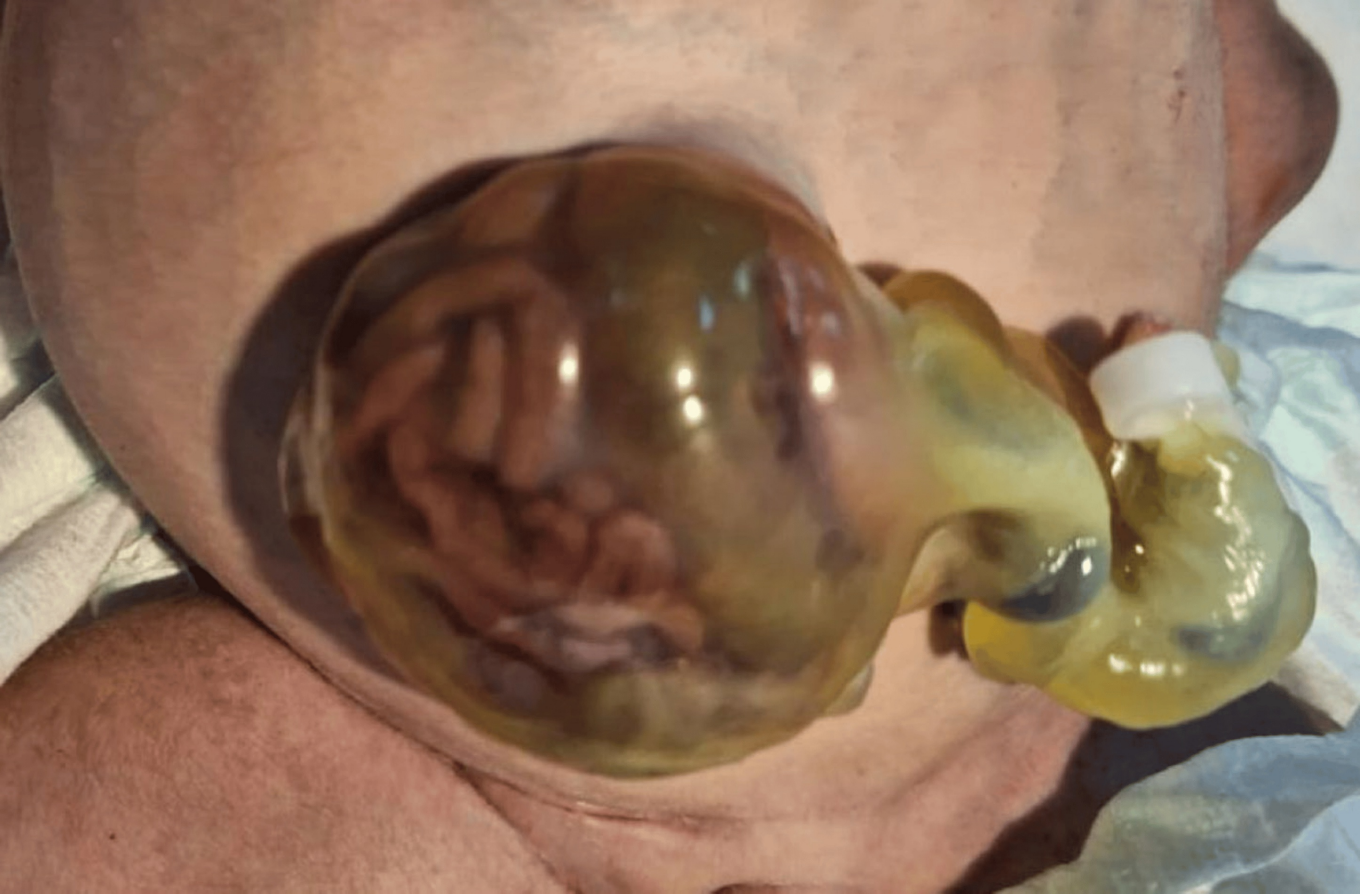
Clinical image of an omphalocele at birth.

**Figure 2:**
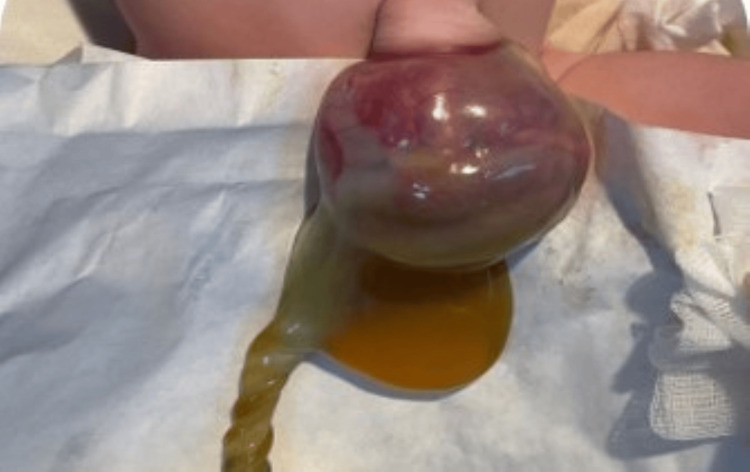
Clinical image of an omphalocele at birth.

**Figure 3:**
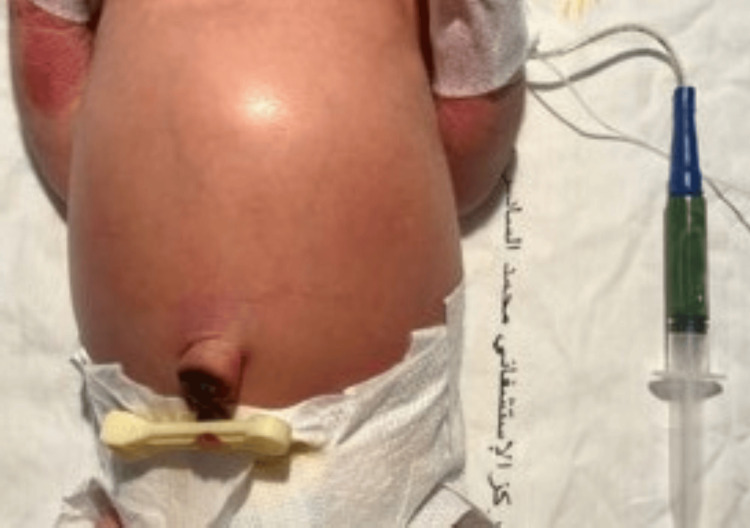
Newborn with abdominal distension and bilious vomiting.

**Figure 4:**
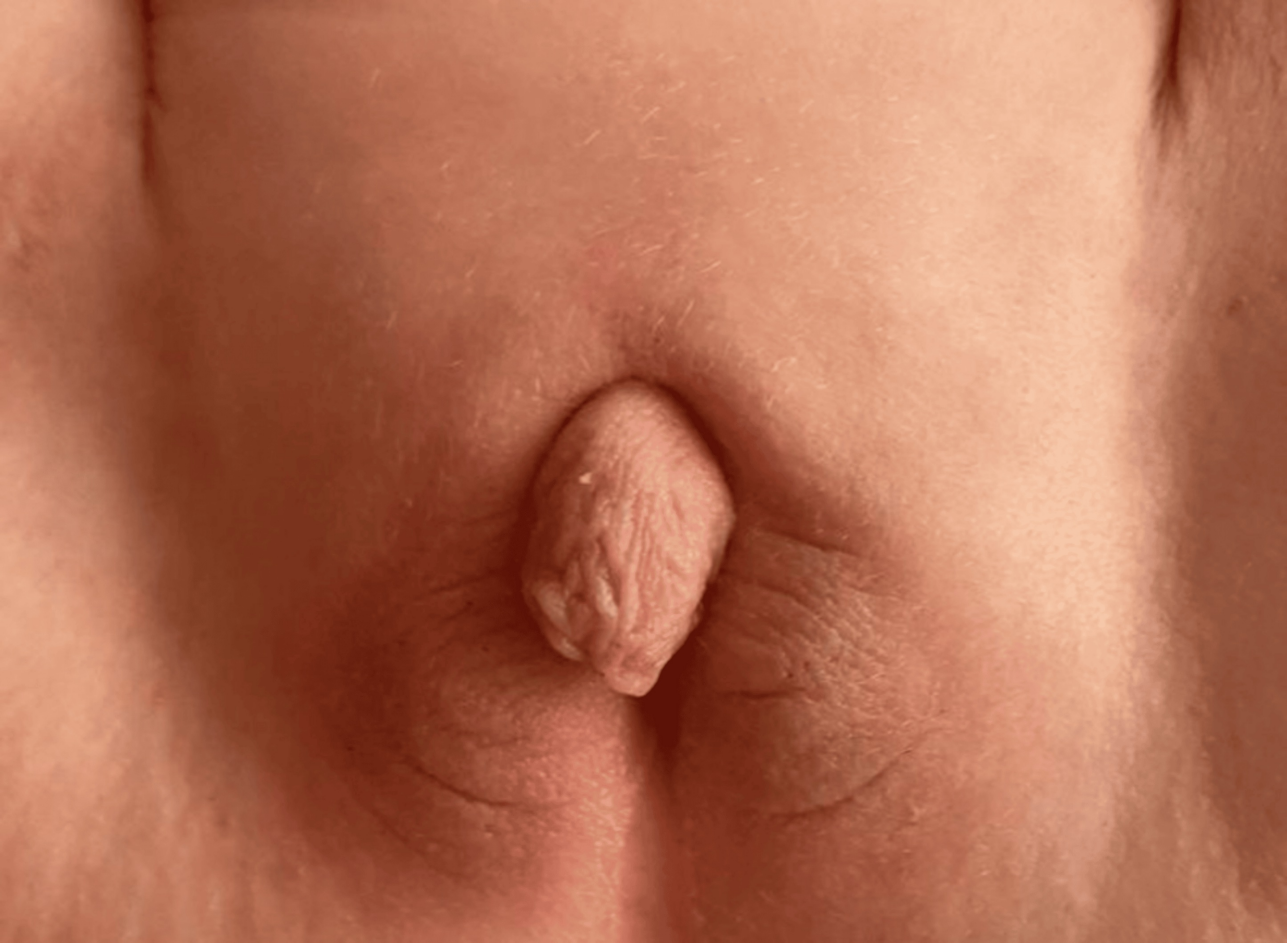
Clinical presentation of ambiguous genitalia in a newborn.

The types of malformations observed were heterogeneous. The most frequent were congenital heart defects (31.25%), followed by anal atresia (10.9%), omphalocele (9.37%) (Figures 1, 2), and neonatal intestinal obstructions (7.8%) (Figure 3). Cases of duodenal atresia (7.8%), disorders of sexual differentiation (6.25%) (Figure 4), congenital diaphragmatic hernia (4.68%), and trisomy 21 (3.12%) were also recorded. Table 1 summarizes the distribution of congenital malformations.

**Table 1:** Distribution of congenital defect types among newborns with malformations.

Congenital defect type	Percentage
Congenital heart defects	31.25% (N = 20)
Anal atresia	10.9% (N = 7)
Omphalocele	9.37% (N = 6)
Intestinal obstruction	7.8% (N = 5)
Duodenal atresia	7.8% (N = 5)
Disorders of sexual differentiation	6.25% (N = 4)
Congenital diaphragmatic hernia	4.68% (N = 3)
Trisomy 21	3.12% (N = 2)
Cleft lip and palate	3.12% (N = 2)
Holoprosencephaly	3.12% (N = 2)
Spina bifida	1.56% (N = 1)
Myelomeningocele	1.56% (N = 1)
Laparoschisis	1.56% (N = 1)
Trisomy 18	1.56% (N = 1)
Trisomy 13	1.56% (N = 1)
Congenital teratoma	1.56% (N = 1)
Anophthalmia	1.56% (N = 1)
Hypertrophic pyloric stenosis	1.56% (N = 1)

**Figure 5:**
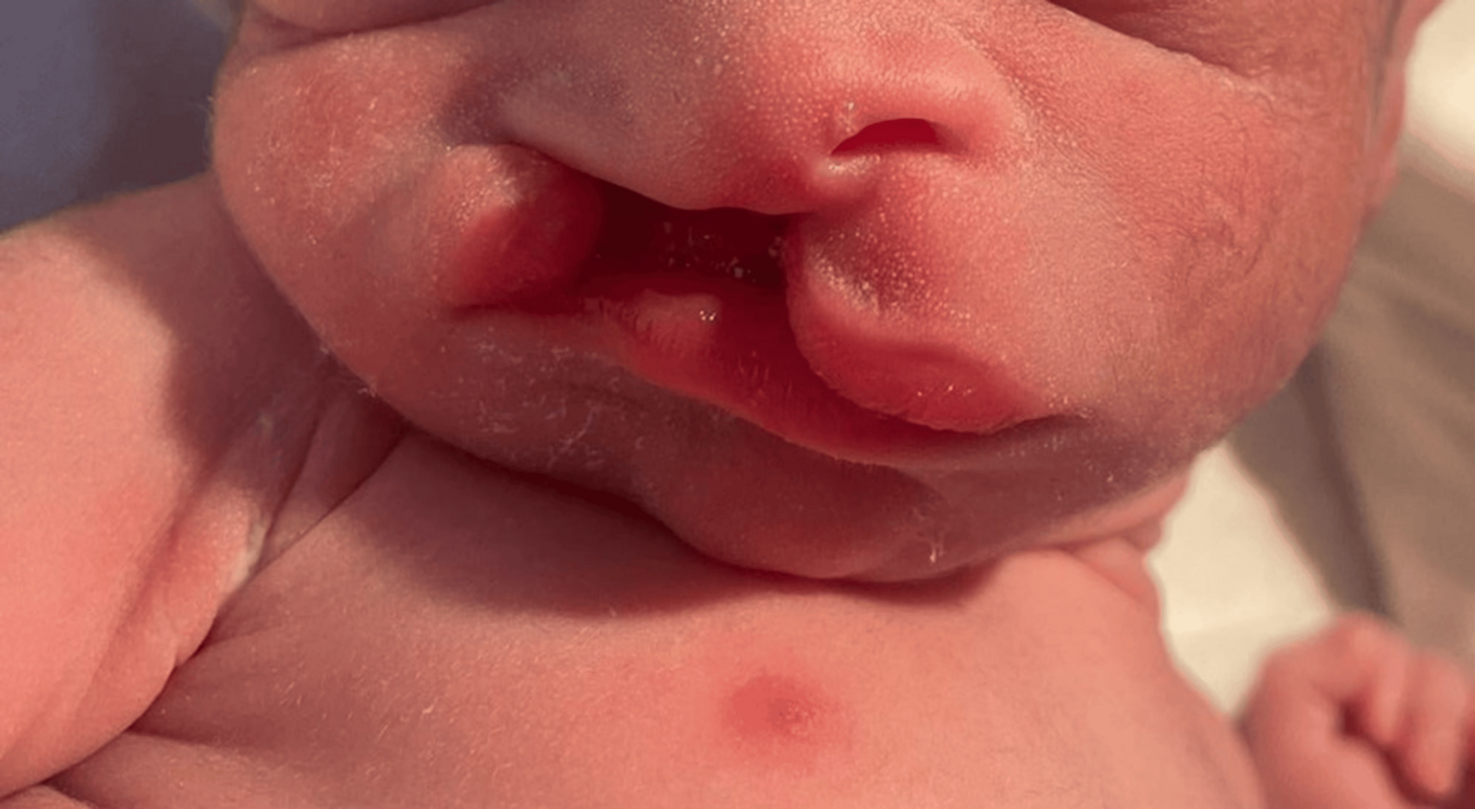
Clinical image of cleft lip and palate in a newborn.

**Figure 6:**
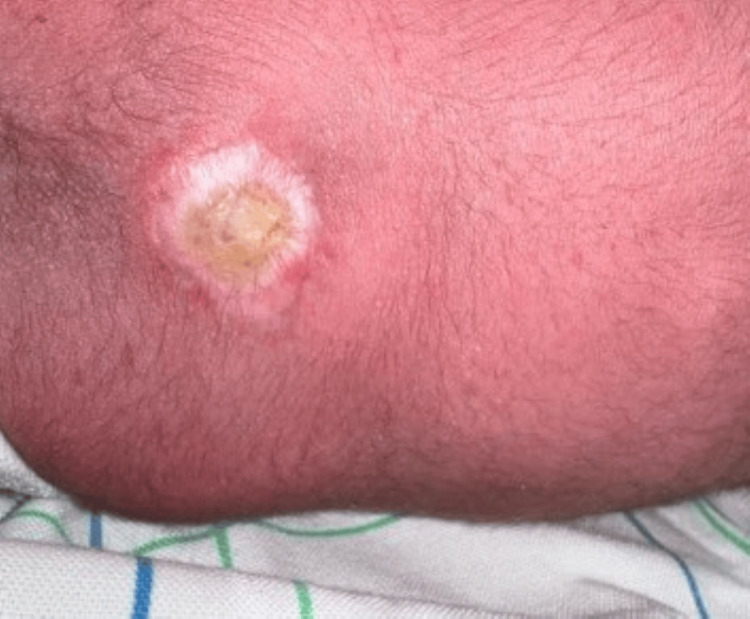
Clinical image of spina bifida occulta in a newborn.

**Figure 7:**
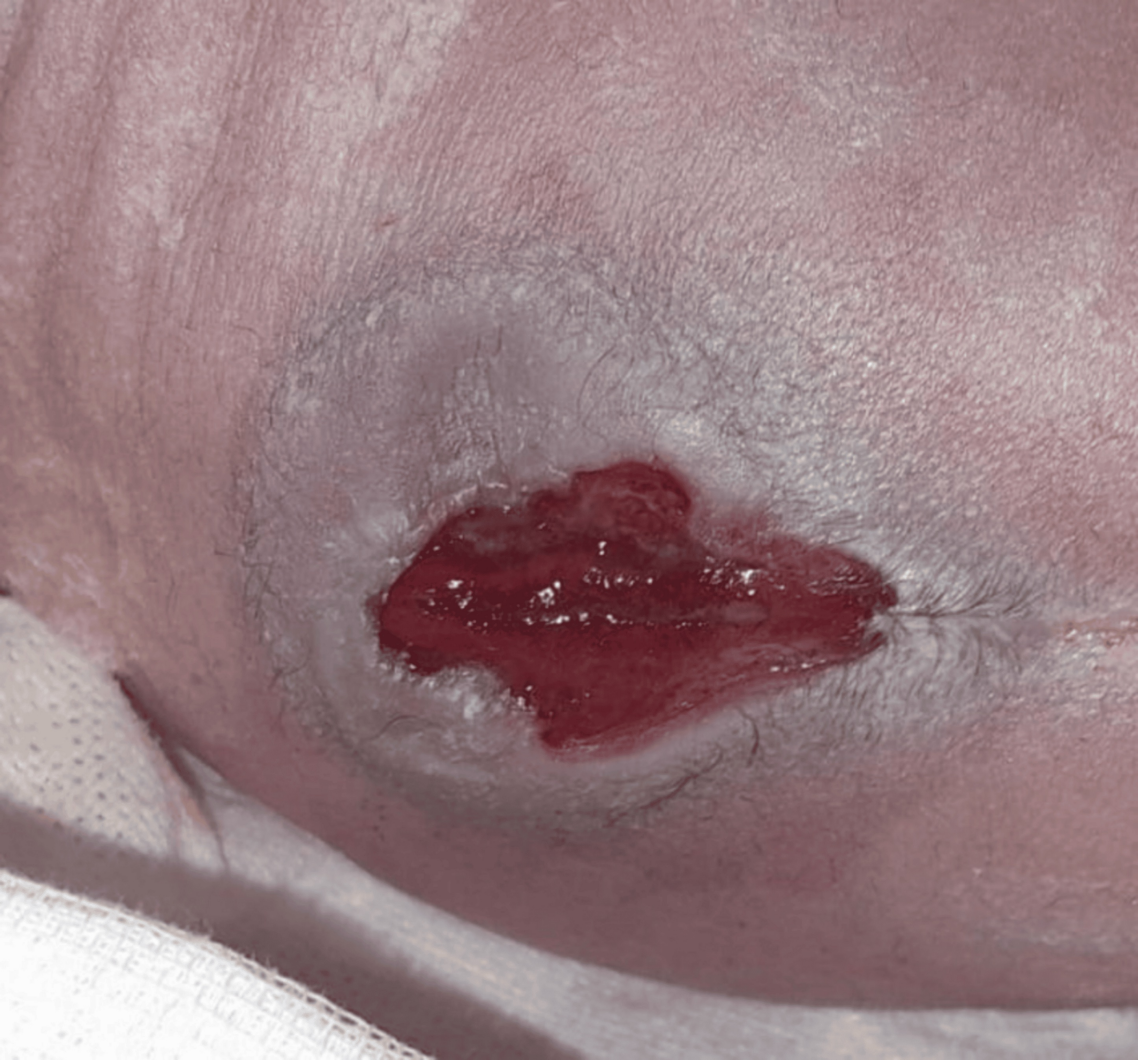
Open myelomeningocele in a newborn.

**Figure 8:**
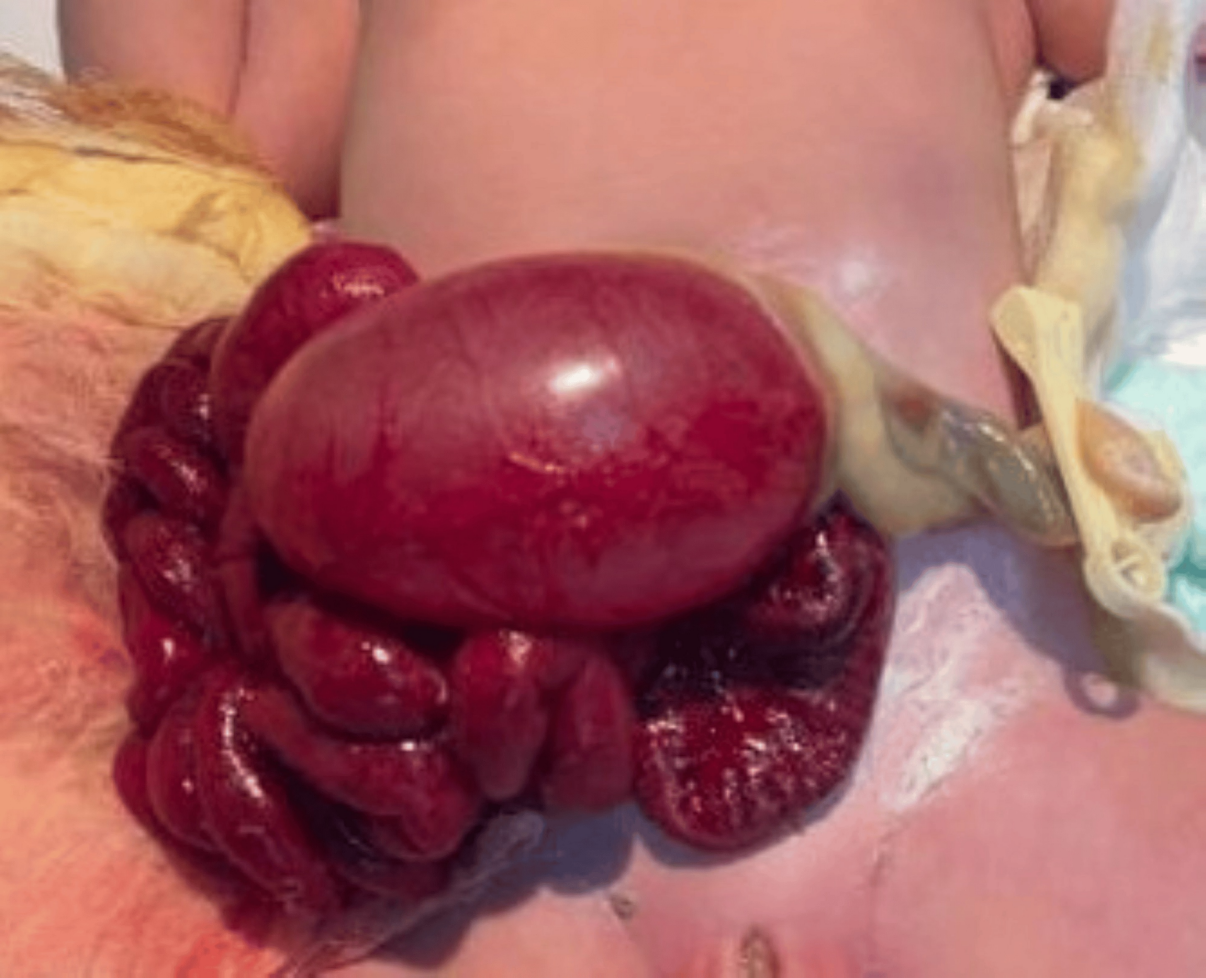
Clinical image of laparoschisis in a newborn.

**Figure 9:**
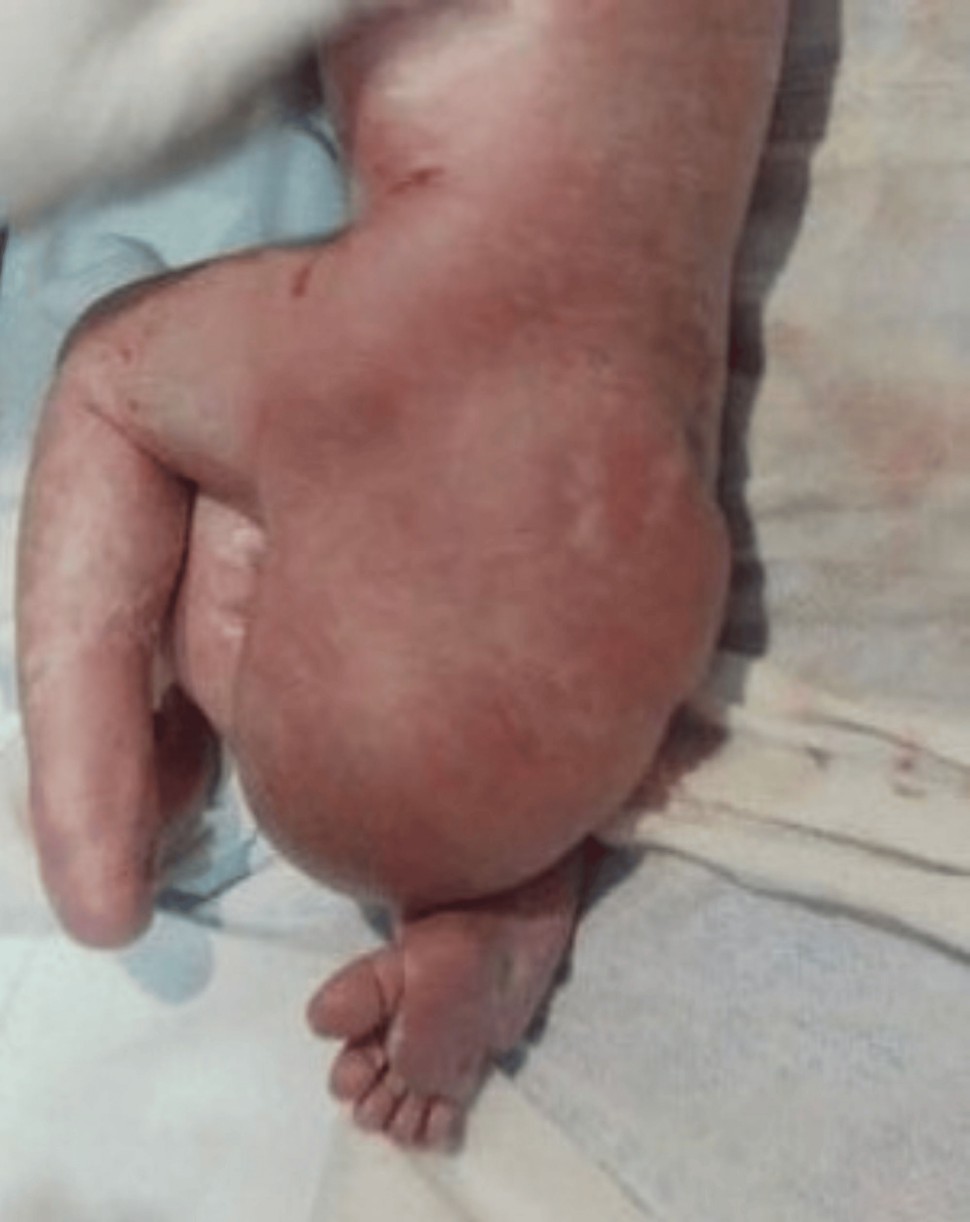
Clinical presentation of a congenital gluteal teratoma.

**Figure 10:**
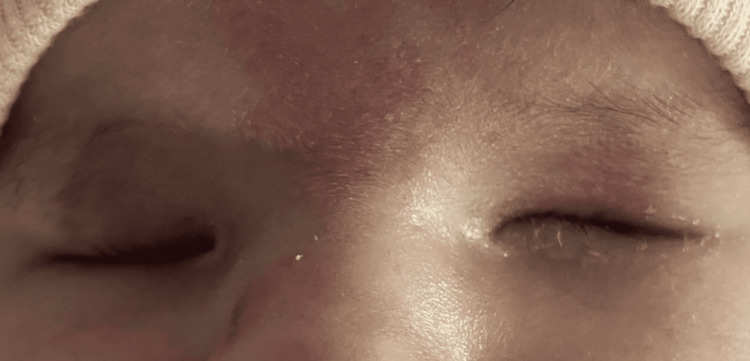
Clinical image of bilateral congenital anophthalmia in a newborn.

Other less frequent anomalies included two cases of cleft lip and palate (Figure 5), two cases of holoprosencephaly, and one case each of spina bifida (Figure 6), myelomeningocele (Figure 7), laparoschisis (Figure 8), trisomy 18 and 13, congenital teratoma (Figure 9), anophthalmia (Figure 10), and hypertrophic pyloric stenosis. Additionally, 13.8% of cases involved multiple congenital anomalies, combining several malformations in the same patient.

With regard to outcomes, the prognosis was variable. A high neonatal mortality rate of 42% was observed. In contrast, 32% of newborns were discharged home after treatment, and 26% were transferred to the pediatric surgery department for further specialized management.

## Discussion

Congenital malformations accounted for 17.3% of neonatal admissions during the study period, a rate higher than those reported in several other studies. For example, a study by Kirby in 2017 in the United States estimated the prevalence of congenital malformations among live-born infants to range generally between 3% and 5% [5]. Additionally, a study led by Feldkamp et al. reported that about 2% of infants have major congenital malformations, excluding common minor anomalies, estimating around 78,000 infants born annually in the United States with serious defects [6]. By comparison, a study conducted in Yopougon, Abidjan (Côte d'Ivoire), reported a frequency of 1.85% [4].

Most malformations occurred in young mothers, consistent with other research. A US cohort study demonstrated a significant association between maternal age 13 to 19 years and various malformations (central nervous system, digestive, and musculoskeletal) [7]. Conversely, multiple and cardiac malformations, more frequent in mothers over 35 years, are often linked to chromosomal abnormalities [8].

In our series, over half the mothers were primiparous, revealing a specific profile in this group. Previous work, such as by Garcon et al. at the State University Hospital of Haiti, found higher congenital malformation prevalence among pauciparous mothers, with a malformation rate around 50% in this group, suggesting moderate parity may also be linked to increased anomaly risk [9].

Consanguinity was identified in 27% of cases, aligning with other studies that consider it a significant factor [10,11]. A Danish study highlighted the importance of considering consanguinity in healthcare, especially where about 7.5% of the population has diverse ethnic origins often associated with consanguineous marriages. The study found that consanguineous couples have a significantly higher risk of having children with congenital malformations compared to non-consanguineous couples. The average population risk in Denmark is estimated at 3%, with autosomal recessive diseases frequently observed among children from these unions [12].

Family history is also critical in risk assessment [13]. Three mothers in our series had gestational diabetes, a condition associated with increased malformation risk; maternal glycemic control disturbances around conception raise the risk, especially well-documented in pregestational diabetes. Several studies, both registry-based and cohort, have shown increased malformation risk with diabetes [14,15].

Regarding parity, multiple studies have examined its impact on congenital malformation risk with contradictory results. Some associate nulliparity with higher risk, suggesting the absence of prior pregnancy may influence certain anomalies, while others report increased risk in multiparous women, possibly related to additional maternal or biological factors with each pregnancy [16,17].

A male predominance with a sex ratio of 1.34 was observed, similar to other studies reporting higher congenital malformation prevalence in males in comparable populations [2,9,18]. The majority of births were at term (92.18%), reflecting data from several African studies [2,19]. However, other research indicates malformation frequency increases with prematurity [20].

In our study, 17.1% of newborns had low birth weight, with an average weight of 2.81 kg (range 1 to 4.5 kg). This average is comparable to studies conducted in Yaoundé (2015-2016) and by Garcon et al. in Haiti (2012-2017) [5,9]. Additionally, a population study by Iliodromiti et al. on 979,912 term singleton births in Scotland (1992-2010) demonstrated a significant association between congenital malformations and birth weight adjusted for gestational age [21].

Congenital heart defects were the most frequent malformations (30.7%), consistent with studies from developed countries where cardiac anomalies predominate [22,23], followed by digestive anomalies. Neural tube defects predominate in studies from Nigeria (2014) and eastern Democratic Republic of Congo [24]. Elsewhere, other malformations such as musculoskeletal anomalies in Yaoundé and polymalformations in Yopougon are more frequent [3,4].

The overall hospital mortality rate observed was high at 42%, comparable to Abidjan’s reported 48.31%, but lower than in the Democratic Republic of Congo, where hospital mortality reached 59.6%. Conversely, a study at the State University Hospital of Haiti reported a lower mortality rate of 30% [4,8,25].

## Conclusions

Congenital malformations constitute a significant challenge in neonatal care due to their clinical severity and potential long-term consequences. In this study, cardiac anomalies were the most frequently observed, consistent with trends reported in other regions. The presence of severe malformations-those threatening immediate survival or leading to lasting functional impairments-emphasizes the need for targeted clinical attention. These findings underscore the importance of implementing comprehensive preventive strategies, including the development of a national congenital malformations registry to enhance epidemiological monitoring and guide public health interventions. Strengthening prenatal care, promoting health education among women of childbearing age, and integrating systematic early prenatal screening may contribute to improved detection, optimized management, and better outcomes for affected newborns.
